# Clinical detection of PSP, not only an “atypical parkinsonism”

**DOI:** 10.1002/alz70857_101937

**Published:** 2025-12-24

**Authors:** Blas Couto, Alonso Morales‐Rivero, Ignacio Flores, Indira Ruth Garcia Cordero, Raul Medina Rioja, Carmela Tartaglia, Gabor G. Kovacs

**Affiliations:** ^1^ Instituto de Neurociencias Cognitivas y Traslacional, INCyT, Buenos Aires, Buenos Aires, Argentina; ^2^ Toronto Western Hospital, University Health Network Memory Clinic, Toronto, ON, Canada; ^3^ Neuroscience Institute, Favaloro Foundation Hospital, Buenos Aires, Argentina; ^4^ Universidad de San Andres, Victoria, Buenos Aires, Argentina; ^5^ Instituto Nacional de Neurología y Neurocirugía “Manuel Velasco Suárez”, Mexico, DF, Mexico; ^6^ Division of Neurology, Toronto Western Hospital, University Health Network, Toronto, ON, Canada; ^7^ Rossy PSP Program, University Health Network and the University of Toronto, Toronto, ON, Canada; ^8^ Tanz Centre for Research in Neurodegenerative Diseases, University of Toronto, Toronto, ON, Canada

## Abstract

Progressive Supranuclear Palsy (PSP) is a neurodegenerative condition that manifest motor, cognitive, and behavioral symptoms. Patients often suffer from early postural instability with falls, diplopia associated with oculomotor dysfunction, apathy, impulsivity, slowness of movement and of thought, as well as memory and language/communication disturbances. With any of those clinical features, patients may seek health care at various medical specialties, not uncommonly, out from neurology clinics. Clinical diagnosis is possible when specific criteria are fulfilled which can only be accomplished by a systematic history taking, time course consideration, and detection of signs at physical neuro‐examination. Up to 4‐years delay in diagnosis has been reported often due to the difficulty in distinguishing PSP findings from those of Parkinson's and Alzheimer's disease (PD and AD) and preventing referral to specialized clinics. In this presentation, the audience will hear about the specific but not hardly detectable clinical features that people with PSP can manifest early in disease course, how those features progress during the mild‐moderate stages of the disease, and how PSP might be distinguishable from PD and AD even after a few clinical encounters.

